# Microbial evaluation and public health implications of urine as alternative therapy in clinical pediatric cases: health implication of urine therapy

**DOI:** 10.4314/pamj.v5i1.56188

**Published:** 2010-05-25

**Authors:** Adenike Adedayo O. Ogunshe, Abosede Oyeyemi Fawole, Victoria Abosede Ajayi

**Affiliations:** 1 Applied Microbiology and Infectious Diseases Unit, Department of Botany and Microbiology, University of Ibadan, Ibadan, Oyo State, Nigeria; 2 Department of Biology, The Polytechnic, Ibadan, Oyo State, Nigeria; 3 Biology and Microbiology Unit, Department of Science Laboratory Technology, Moshood Abiola Polytechnic, Abeokuta, Ogun State, Nigeria

**Keywords:** Alternative medicine, Antibiotic resistance, Convulsion, Cultural behaviour, Infant mortality, Pediatic, Urine therapy, Nigeria

## Abstract

**Background:**

Cultural means of pediatric treatment during ill health is a mainstay in Africa, and though urine has been known to contain enteric pathogens, urine therapy is still culturally applicable in some health conditions and also advocated as alternative therapy. The study therefore, is to evaluate the microbial contents and safety of urine.

**Methods:**

Urinary bacteria from cows and healthy children aged 5-11 years were identified by conventional phenotypic methods and antimicrobial susceptibility testing was performed using modified agar disc and well-diffusion methods.

**Results:**

A total of 116 bacterial isolates (n = 77 children; n = 39 cows) were identified as Bacillus (10.4%; 5.1%)), Staphylococcus (2.6%; 2.6%), Citrobacter (3.9%; 12.8%), Escherichia coli (36.4%; 23.1%), Klebsiella (7.8%; 12.8%), Proteus (18.2%; 23.1%), Pseudomonas (9.1%; 2.6%), Salmonella (3.9%; 5.1%) and Shigella (7.8%; 12.8%) spp. Antibiotic resistance rates of the Gram-positive bacteria were high (50.0100%), except in Bacillus strains against chloramphenicol, gentamicin and tetracycline (14.3%), while higher resistance rates were recorded among the Gram-negative bacteria except in Citrobacter (0.0%) and Proteus (8.5%) spp. against gentamicin and tetracycline respectively. The Gram-negative bacteria from ito malu (cow urine) were more resistant bacteria except in Citrobacter (20.0%) and Shigella spp. (0.0%) against tetracycline and Proteus spp. (11.1%), (22.2%) against amoxicillin and tetracycline respectively. Multiple antibiotic resistance (MAR) rates recorded in children urinal bacterial species were 37.5-100% (Gram-positive) and 12.5-100% (Gram-negative), while MAR among the cow urinal bacteria was 12.5-75.0% (Gram-positive) and 25.0-100% (Gram-negative). Similar higher resistance rates were also recorded among the Gram-negative bacterial species from urine specimens against the pediatric antibiotic suspensions.

**Conclusion:**

The study reported presence of multiple antibiotic-resistant indicator bacteria in human urine and ito malu used as alternative remedy in pediatric health conditions like febrile convulsion.

## Background

There are various family behavioral practices in medicine and health but some of the traditional healing practices in Africa include herbal therapies [1] and other forms of alternative therapies which are non-clinical. Traditional home remedies are still commonly used in childhood ill-health conditions like convulsions in Nigeria, and the administered remedy depends on the practices of the community [2]. Based on certain traditional points of view, consumption of *ito malu* (cow urine) and human urine is a common form of traditional remedy for convulsion and related health conditions in children in some cultural settings in Nigeria. Similarly, current propaganda on urine therapy has also started to rise. The practice of using urine in traditional medicine and healthcare in parts of Nigeria are scantily reported but it is known that some parents use human and cow urine as a form of folk medicine in paediatric ill health, most especially in cases of febrile convulsion; the most common type of convulsion in childhood, which is usually frightening to parents [3] and which according to the National Institute of Health Consensus Statement [4] is defined as an event in infancy or childhood, usually occurring between 3 months and 5 years of age, and associated with fever but without evidence of intracranial infection or defined cause for the seizure.

Lack of proper delivery of health care services in the country has strongly supported the increase in any form of alternative therapy in the country, including the advocacy for urine therapy. Kafaru [5] stated that many of the Nigerian older people used human urine in serious cases of ill-health such as wound sores, diabetes and even when poison was suspected; while cow urine is mixed with herbal preparation for treating convulsion in babies. Shodipe [6] reported that in traditional treatment of sickle cell anemia, grinded leaves of tobacco (*Nicotiana tabacum*) are soaked in six pints of cow urine and six bottles of aromatic schnapps gin, then 1 desert spoon of rock salts is added to the mixture in a big bottle corked and kept for 2 days. Adodo [7] also reported that urine therapy can cure cancer. Narrating a personal experience in using auto-urine for therapy, Kafaru [5] reported that in 1980, when she was bleeding during a four-month pregnancy, she drank only urine and water to stop the bleeding after six hours. She was of the opinion that urine therapy is the most curative, easy-to-use and most cost effective.

Previous studies however, had reported that urine usually contained many enteric bacteria and that most urinary tract infections (UTIs) are caused by enteric bacteria [8-14]. Possible bacterial pathogens that are found in urine include enterococci, such as Enterococcus *faecalis, Bacillus, Staphylococcus saprophyticus*, haemolytic streptococci, such as *Streptococcus agalactiae, Citrobacter spp., E. coli, Enterobacter aerogenes, Enterobacter cloacae*, coagulase-negative staphylococci, Proteus spp., such as *Proteus mirabilis, Pseudomonas aeruginosa*, Klebsiella spp., such as *Klebsiella pneumoniae, Klebsiella oxytoca, Acinetobacter* species and diptheroids, while yeasts like *Candida* spp. may also be found in the female urethra, caused by contamination of the urine with the skin. Commensals including staphylococci and Mycobacterium smegmatis may also occur as specimen is being collected [11, 15-16]. It is quite true that most African countries have traditional means of curing clinical conditions; however, it is very fundamental and necessary that adequate measures are taken to address cultural health situations that are known to pose great risks, especially to children. It is thereby necessary that treatment of infectious or non-infectious diseases, especially of public health importance using traditional remedies should be supported by scientific evidence. This present study therefore, tries to provide information from the microbiological point of view, on the public health significance of urine of children and cows as alternative therapy in pediatric cases, and to serve as a forum for the exchange of ideas and clarification on this speculated traditional means of curing pediatric related ill health conditions like convulsion.

## Methods

### Samples’ collection and analyses

Microbiological cultures were performed on aseptically collected 100 early morning midstream urine specimens of healthy children (53 female, 47 male), aged 5-11 years in two state capitals of Nigeria, Abeokuta (Ogun state) and Ibadan (Oyo state). The subjects were not on antibiotic therapy within 3 months prior to the period of specimens’ collection. The collection process was without any inclusion or exclusion criteria, such as acute voiding symptoms, significant bacteriuria with growth of at least 10 colony-forming units/ml urine, leukocyturia > 50/ μl, underlying renal diseases, anatomic abnormalities of the urinary tract, like in the study of Pape et al. [11]. Consenting parents were trained on how to collect freshly-voided

midstream urine specimens of their children. The cow urine specimens were personally collected from Bodija and Moniya abattoirs, after obtaining the consents of the personnel in charge. All the urine specimens were cultured within 4 hours on same days of collection.

### Isolation and characterization of bacterial species from the urine specimen

Urine culture were determined on plate count agar (PCA; LAB M), eosin methylene blue agar (EMB), MacConkey (MCC; LAB M) agar and cystein lactose electrolyte deficient (CLED; LAB M) agar at 350 degree Celsius for 24-48 hours. Different colonies on culture plates having ≥1.0 x 104 cfu ml-1 were randomly selected and colonies sub cultured on CLED and MacConkey agars. Pure bacterial isolates were identified to species level by the standard taxonomic protocol of the laboratory, based on the phenotypic profiles, including their cultural and microscopic morphologies as well as basic biochemical procedures [17].

### Antibiotic susceptibility determination (discs)

The antimicrobial susceptibility testing on the bacterial isolates was performed using the disc diffusion method. The antibiotics included in the determination of antibiotic resistance in this study were the range of antibiotic classes commonly imported into the country (Amoxicillin (25μg), Augmentin (30μg), Cloxacillin (5μg), Erythromycin (5μg), Chloramphenicol (30μg), Cotrimoxazole (25μg), Gentamicin (10μg) and Tetracycline (10μg), while for the Gram-negative bacterial strains were- Penicillin (10μg), Clindamycin (20μg), Gentamicin (10μg), Fusidic acid (10μg), Erythromycin (5 μg), Trimethoprim (25μg), Sulphamethaxazole (25μg) and Tetracycline (10μg)) and routinely used in the treatment of UTIs. Zones of inhibition were measured and the diameter recorded in millimeter. Zones less than 10.0 mm in diameter or absence of inhibition zones were recorded as resistant (negative) [18-19]. The antibiotic discs were obtained from ABTEK Biologicals Ltd. (Liverpool, UK).

### Antibiotic susceptibility determination (pediatric suspensions)

The twenty one pediatric antibiotic suspensions, ampicillin (Emicillin; 125mg/5ml), amoxicillin/Clavulanic acid (Fleming; 228.5 mg/5ml), metronidazole (Ioxagyl; 200 mg/5ml), cotrimoxazole (Septrin; 240 mg/5ml), cefuroxime axetil (Zinnat; 125 mg/5ml), amoxicillin (Amaxin; 125 mg/5ml), Fluiloxacillin (Floxapen; 125 mg/5ml), azithromycin (Zithromax; 200 mg/5ml), ampicillin / cloxacillin (Jawaclox; 250 mg/5ml), Erythromycin (Erythrokid; 250 mg/5ml), Cefalerin (Cefamor; 125 mg/5ml), Erythromycin (Etocin; 200 mg/5ml), Erythromycin (Throtal; 125 mg/5ml), Cotrimoxazole B.P. (Loxaprim; 240 mg/5ml), Cotrimoxazole (Rancotrim; 240 mg/5ml), Cotrimoxazole (200 mg/5ml), Chloramphenicol (Clofencol; 125 mg/ 5ml), Cefaclor (Vercef; 125 mg/5ml), ampicillin + cloxacillin (Emzoclox; 250 mg/5ml), Sulfamethoxazole + Trimethoprim (Bactrim; 240 mg/5ml) and Cefadroxil (Odoxil DS; 250 mg/5ml) were assayed for in this study using the modified [20] agar well-diffusion method of Tagg et al. [21]. Sterile Mueller-Hinton agar was poured into sterile Petri dishes and allowed to set. Wells about 6.0 mm were bored into the agar followed by surface sterilization of the agar plates by flaming. The entire surface of each of the sterile Mueller-Hinton agar plates was then streaked with each bacterial isolate using sterile swab sticks. The plates were left for about 10 minutes before aseptically dispensing the pediatric antibiotic suspensions (antibiotic powder dissolved in recommended volume of sterile distilled water) into the agar wells. The plates were then incubated in upright position at 350C for 24 hours. Zones of inhibition were measured and the diameter recorded in millimeter. Although, the interpretations of the inhibitory zones of antimicrobial agents like antibiotics vary, however, just like some earlier workers, zones of inhibition less than 10.0 mm in diameter or absence of inhibition zones were recorded as resistant (negative) [18, 19].

## Results

The pH of the children urine specimens were between 5.5 and 14.0 (Abeokuta) and 6.0 and 13.0 (Ibadan) but more of the urine specimens had pH of 6.5 (14.0%), 7.0 (21.0%) and 6.0 (27.0%). Other pH values were 7.5 (7.0%), 8.0 (9.0%), 8.5 (5.0%), 9.0 (5.0%), 9.5 (3.0%), 12 and 14 (1.0%). The pH of the cow urine samples were 7.5 (12.1%), 8.0 (54.5%), 8.5 (24.2%) and 9.0 (9.1%). Total colony counts of the urine specimens were between 1.0 x 102 and 1.2 x 108 cfu ml-1 on CLED; 1.5 x 102 and 8 x 107 cfu ml-1 on MCC; 2.2 x 103 and 1.2 x 105 cfu ml-1 on EMB agar plates. The counts on PCA were too numerous to count on most of the culture plates at dilutions between 10-2 and 10-4 while at dilutions of 10-5 to 10-6, countable colonies of 1.4 x106 - 1.2 x 107 cfu ml-1 were obtained.

Apart from fungal colonies that were obtained from the urine specimens, 77 bacterial strains were randomly isolated from 100 children urine specimens, while 39 bacterial strains were isolated from 33 urine specimens of cows. The identified Gram-positive bacterial isolates obtained from the children urine specimens were Bacillus 8 (10.4%) and Staphylococcus 2 (2.6%) species, while Gram-negative bacterial species were Citrobacter 3 (3.9%), Escherichia coli 28 (36.4%), Klebsiella 6 (7.8%), Proteus 14 (18.2%), Pseudomonas 7 (9.1%), Salmonella 3 (3.9%) and Shigella 6 (7.8%) (Table 1). Only Salmonella, Shigella and Staphylococcus species were not obtained from Abeokuta urine specimens. As shown in Table 2, the bacterial species from the urine specimens of cows were Bacillus 2 (5.1%), Staphylococcus 1 (2.6%), Citrobacter 5 (12.8%), Escherichia coli 9 (23.1%), Klebsiella 5 (12.8%), Proteus 9 (23.1%), Pseudomonas 1 (2.6%), Salmonella 2 (5.1%) and Shigella 5 (12.8%).

In this study, the antibiotic resistance patterns of the bacterial isolates from the urine specimens of children (antibiotic discs) indicated that the antibiotic resistance of the Gram-positive bacterial species (Bacillus and Staphylococcus) were high (50.0-100%), except in Bacillus strains against chloramphenicol, gentamicin and tetracycline (14.3%). Higher resistance rates were also recorded among the Gram-negative bacteria against the test antibiotics except in Citrobacter (0.0%) and Proteus (21.4%) towards gentamicin. Multiple antibiotic resistance recorded among the Gram-positive bacterial species were 37.5-87.5%, while 12.5-100% MAR were recorded among the Gram-negative bacterial species from the urine specimens of children. At least, thirteen of the bacterial strains had total resistance (100%), while only maximum of five strains had total (100%) susceptibility towards all the test antibiotics (Table 1).

The Bacillus strains from the urine specimens of cows were more resistant to the test antibiotics (50.0-231 100%), except in chloramphenicol, in which no resistance was recorded. Similarly, the resistance patterns of the Gram-negative bacterial species from urine of cows were more resistant towards the test antibiotics (40.0-100%), except in Citrobacter, Klebsiella and Proteus against gentamicin (20.0%; 20.0%, 11.1%) and Citrobacter and Proteus against tetracycline (20.0%; 22.2%) respectively. No resistance was recorded among the Shigella strains against tetracycline. The MAR recorded among the bacterial species from the urine specimens of cows in this study was between 12.5 and 100%. Six of the bacterial strains from urine specimens of cows had total (100%) susceptibility, while only two strains had total (100%) resistance towards all the test antibiotics (Table 2).

As shown in Table 3, all the Bacillus and Staphylococcus strains from children urine were moderately or highly resistant to the pediatric antibiotic suspensions except in ampicillin/ cloxacillin (Emzoclox) and flucloxacillin (Floxapen) (12.5%); azithromycin (Zithromax) cotrimoxazole (Ranotrim), erythromycin (Erythrokid) and sulfamethoxazole/trimethoprim (Bactrim) (25.0%) to which the Bacillus strains were more susceptible. Total susceptibility was recorded in Bacillus strains towards ampicillin/cloxacillin (Jawaclox), in Staphylococcus strains towards amoxicillin/ clavulanic acid (Fleming), ampicillin/cloxacillin (Jawaclox) and azithromycin (Zithromax).

Relatively higher resistance rates were recorded by the bacterial species from urine of cows towards the test paediatric antibiotics although total (0.0%) susceptibility were exhibited towards few of the bacterial strains by some paediatric antibiotics [Amoxicillin/clavunlanic acid (Fleming), Ampicillin (Emcillin), ampicillin\cloxacillin (Emzoclox), ampicillin\cloxacillin (Jawaclox), Azithromycin (Zithromax), Cefaclor (Vercef), Cefalexin (Cefamor), Cefuroxine (Amaxin), Chloramphenicol (Clofencol), cotrimoxazole (Septrin), Cotrimoxazole B.P (Loxaprim), cotrimoxazole (Rancotrim), erythromycin (Erythrokid), erythromycin (Etocin), Metronidazole (Loxagyl), sulfamethoxazole/trimethoprim (Bactrim) (Table 4).

Multiple antibiotic resistance patterns were also exhibited by the Gram positive bacterial strains from the urine of children (Bacillus; 28.6 – 61.9%) and (Staphylococcus; 38.1 – 81.0%) against the pediatric antibiotic suspensions (Table 3), while as high as 81.0% MAR was also exhibited by the bacterial species from cows towards the pediatric antibiotic suspensions (Table 4).

**Table 1:** Antibiotic resistance patterns of isolated bacterial flora from urine samples of children using antibiotic discs

	% Antibiotic resistance (μg) / disc								
**Isolates**	**AUG**	**AMX**	**ERY**	**TET**	**CXC**	**GEN**	**COT**	**CHL**	**MAR**
*Bacillus* spp. [8]	87.5	100	57.1	14.3	100	14.3	57.1	14.3	(37.5 – 87.5)
*Staphylococcus* sp. [2]	50.0	50.0	50.0	50.0	50.0	50.0	50.0	50.0	(100.0)1*1±
	**PEN**	**CLN**	**GEN**	**FUS**	**ERY**	**TRM**	**SMX**	**TET**	
*Citrobacter* spp. [3]	100	100	0.0	100	100	33.3	33.3	33.3	(50.0 – 75.0)
*E. coli* [28]	85.7	60.7	28.6	60.7	67.9	60.7	67.9	46.4	(12.5–100)2*5±
*Klebsiella* spp. [6]	100	66.7	33.3	50.0	66.7	100	83.3	33.3	(37.5 – 87.5)
*Proteus* spp. [14]	71.4	57.1	21.4	50.0	50.0	64.3	64.3	8.5	(12.5–100)2*3±
*Pseudomonas* spp. [7]	100	87.5	28.6	71.4	71.4	71.4	71.4	42.9	(37.5 -100) 2±
*Salmonella* spp. [3]	100	66.7	66.7	66.7	66.7	100	100	33.3	(37.5 – 100.0)1±
*Shigella* spp. [6]	100	66.7	16.6	33.3	83.3	66.7	33.3	33.3	(25.0 -100)1±

AUG: Augmentin, AMX: Amoxycillin, ERY: Erythromycin, TET: Tetracycline, CXC: Cloxacillin, GEN: Gentamicin, COT: Cotrimoxazole, CHL: Chloramphenicol, PEN: Penicillin, CLN: Clindamycin, GEN: Gentamicin, FUS: Fusidic acid, ERY: Erythromycin, TRM: Trimethoprim, SMX: Sulphamethaxazole, TET: Tetracycline, *: total susceptibility, ± : total resistance. [X] Reference in the text

**Table 2:** Antibiotic resistance of isolated bacterial flora from urine samples of cows using antibiotic discs

	% Antibiotic resistance (μg) / disc								
**Isolates**	**AUG**	**AMX**	**ERY**	**TET**	**CXC**	**GEN**	**COT**	**CHL**	**MAR**
*Bacillus* spp. [2]	100	100	100	100	100	50.0	50.0	0.0	(75.0)
*Staphylococcus* sp. [1]	**0.0**	0.0	0.0	100	0.0	0.0	0.0	0.0	(12.5)
									
	**PEN**	**CLN**	**GEN**	**FUS**	**ERY**	**TRM**	**SMX**	**TET**	
*Citrobacter* spp. [5]	80.0	80.0	20.0	80.0	80.0	60.0	40.0	20.0	(50.0–100.0) 1*1±
*E. coli* [9]	100	88.9	33.3	77.8	100	66.7	33.3	55.6	(50.0–100.0) 2*
*Klebsiella* spp. [5]	80.0	100	20.0	100	100	100	80.0	60.0	(62.5–100.0) 1*
*Proteus* spp. [9]	88.9	55.6	11.1	44.4	55.6	100	88.9	22.2	(37.5–100.0) 1*
*Pseudomonas* spp. [1]	100	100	100	100	100	100	100	100	(100.0) 1*
*Salmonella* spp. [2]	100	100	100	50.0	100	50.0	100	50.0	(62.5–100.0) 1±
*Shigella* spp. [5]	80.0	80.0	40.0	40.0	60.0	80.0	60.0	0.0	(25.0- 87.5)

AUG: Augmentin, AMX: Amoxycillin, ERY: Erythromycin, TET: Tetracycline, CXC: Cloxacillin, GEN: Gentamicin, COT: Cotrimoxazole, CHL: Chloramphenicol, PEN: Penicillin, CLN: Clindamycin, GEN: Gentamicin, FUS: Fusidic acid, ERY: Erythromycin, TRM: Trimethoprim, SMX: Sulphamethaxazole, TET: Tetracycline, *: total susceptibility, ±: total resistance. [X] Reference in the text

**Table 3:** Antibiotic resistance pattern of isolated bacterial flora from urine samples of children using pediatric oral suspensions (mg/ml)

	% Antibiotic resistance							
**Antibiotic suspensions mg/ml)**	***B. ce r*[8]**	***Staph*[2]**	***Citro*[3]**	***E. coli*[27]**	***Kleb*[6]**	***Prot*[14]**	***Salm*[3]**	***Shig*[6]**
*Amoxicillin (Amaxin)*	50.0	50.0	100.0	35.7	50.0	35.7	33.3	50.0
*Amoxicillin / clavunlanic acid (Fleming)*	50.0	0.0	100.0	28.6	66.7	35.7	33.3	33.3
Ampicillin (Emcillin)	75	50	33.3	28.6	50.0	14.3	66.7	33.3
Ampicillin/ cloxacillin (Emzoclox)	12.5	50.0	66.7	14.3	33.3	21.4	0.0	16.7
*Ampicillin / cloxacillin (Jawaclox)*	0.0	0.0	100	28.6	50.0	28.6	33.3	33.3
*Azithromycin (Zithromax)*	25.0	0.0	66.7	25.0	50.0	28.6	33.3	33.3
*Cefaclor (Vercef)*	50.0	50.0	66.7	53.6	50.0	35.7	33.3	16.7
*Cefadroxil (Odoxil DS)*	75.0	50.0	66.7	71.4	33.3	85.7	33.3	66.7
*Cefalexin (Cefamor)*	87.5	50.0	100	60.7	33.3	78.6	33.3	33.3
*Cefuroxine (Amaxin)*	50.0	50.0	66.7	67.9	83.3	57.1	66.7	50.0
*Chloramphenicol (Clofencol)*	100	100	100	60.7	100	71.4	66.7	66.7
Cotrimoxazole (Septrin)	62.5	100	33.3	28.6	33.3	0.0	33.3	0.0
Cotrimoxazole (B.P (Loxaprim)	50.0	100	33.3	25.0	50.0	42.5	66.7	66.7
Cotrimoxazole (Ranotrim)	25.0	50.0	66.7	14.3	66.7	35.7	33.3	16.7
Cotrimoxazole	50.0	100	100	17.9	66.7	28.6	33.3	50.0
Erythromycin (Erythrokid)	25.0	50.0	66.7	35.7	33.3	42.9	33.3	33.3
Erythromycin (Etocin)	62.5	50.0	100	32.1	66.7	64.3	0.0	50.0
Erythromycin (Throtal)	62.5	100	100	78.6	100	85.7	33.3	100
Flucloxacillin (Floxapen)	12.5	50.0	33.3	32.1	33.3	42.9	33.3	66.7
Metronidazole (Loxagyl)	62.5	100	100	78.6	66.7	71.4	66.7	66.7
Sulfamethoxazole/Trimethoprim (Bactrim)	25.0	100	66.7	17.9	50.0	42.9	33.3	66.7
MAR	28.6 - 61.9	38.1-	57.1 - 100	52.4 - 61.9	38.1 -	14.3 - 81.0	19.0 - 85.7	28.6 - 81.0

Ant: antibiotics suspensions, *B. cer: Bacillus cereus*, Staph: *Staphylococcus aureus*, Citro: *Citrobacter*, E. coli: *Escherichia coli*, Kleb: *Klebsiella*, Prot: *Proteus*, Salm: *Salmonella*, Shig: *Shigella*, MAR: Multiple Antibiotic Resistance. [X] Reference in the text

**Table 4:** Antibiotic resistance pattern of isolated bacterial flora from urine samples of cows using pediatric oral suspensions (mg/ml)

	% Antibiotic resistance							
**Antibiotic suspensions mg/ml)**	***B. ce r*[2]**	***Staph*[1]**	***Citro*[5]**	***E. coli*[9]**	***Kleb*[5]**	***Prot*[9]**	***Pseud [1]***	***Salm [2]***	**Shig [5]**
*Amoxicillin (Amaxin)*	100	100	100	66.7	60.0	66.7	100	50.0	20.0
*Amoxicillin / clavunlanic acid (Fleming)*	100	0.0	80.0	66.7	80.0	44.4	0.0	100	80.0
Ampicillin (Emcillin)	100	0.0	100	77.8	40.0	55.6	0.0	100	60.0
Ampicillin/ cloxacillin (Emzoclox)	50.0	100	20.0	22.2	40.0	11.1	100	0.0	60.0
*Ampicillin / cloxacillin (Jawaclox)*	100	0.0	80.0	77.8	80.0	44.4	100	50.0	80.0
*Azithromycin (Zithromax)*	50.0	0.0	20.0	44.4	20.0	66.7	0.0	0.0	40.0
*Cefaclor (Vercef)*	0.0	100	20.0	55.6	60.0	33.3	0.0	0.0	20.0
*Cefadroxil (Odoxil DS)*	100	100	20.0	44.4	60.0	11.1	100	50.0	40.0
*Cefalexin (Cefamor)*	0.0	0.0	40.0	55.6	80.0	55.6	0.0	50.0	60.0
*Cefuroxine (Amaxin)*	50.0	0.0	40.0	33.3	60.0	77.8	100	50.0	100
*Chloramphenicol (Clofencol)*	100	100	60.0	55.6	80.0	44.4	0.0	100	40.0
Cotrimoxazole (Septrin)	0.0	0.0	20.0	44.4	0.0	66.7	0.0	50.0	100
Cotrimoxazole (B.P (Loxaprim)	100	100	40.0	55.6	80.0	44.4	0.0	50.0	20.0
Cotrimoxazole (Ranotrim)	50.0	100	40.0	33.3	60.0	22.2	100	0.0	0.0
Cotrimoxazole	100	100	60.0	55.6	80.0	33.3	100	50.0	40.0
Erythromycin (Erythrokid)	50.0	0.0	20.0	44.4	40.0	11.1	100	50.0	60.0
Erythromycin (Etocin)	0.0	100	60.0	55.6	40.0	11.1	100	50.0	60.0
Erythromycin (Throtal)	50.0	100	60.0	77.8	80.0	88.9	100	100	80.0
Flucloxacillin (Floxapen)	100	100	80.0	100	80.0	22.2	100	50.0	60.0
Metronidazole (Loxagyl)	100	0.0	80.0	44.4	60.0	66.7	0.0	50.0	100
Sulfamethoxazole/Trimethoprim (Bactrim)	50.0	100	40.0	44.4	60.0	55.6	0.0	50.0	40.0
MAR	57.1 - 61.9	57.1	19.0 - 61.9	28.6 - 81. 0	42.9 - 81.0	23.8 - 61.9	52.4	47.6 - 52.4	23.8 - 71.4

Ant: antibiotics suspensions; *B. cer: Bacillus cereus*; Staph: *Staphylococcus*; Citro: *Citrobacter*; Kleb: *Klebsiella*; Prot: *Proteus*; Salm: *Salmonella*; Shig: *Shigella*; Pseud: *Pseudomonas*; MAR: Multiple Antibiotic Resistance. [X] Reference in the text

## Discussion

Febrile seizures, which carry a good prognosis around the world are associated with a relatively high mortality and morbidity in Africa and other courtiers of the world [22,23]. Convulsion among children between six months and five years is a major contributor to childhood mortality in less-developed societies, especially in sub-Saharan Africa [24]. In Nigeria, this has been attributed to the administration of some indigenous concoctions before the children are brought to hospital [25-27]. The traditional concoctions used in the treatment of convulsion vary with cultural practices [26], and based on the parental fears in cases of seizures, as well as accumulating epidemiological evidence indicating that febrile seizures are the most common recognized antecedent for epilepsy in childhood [28-30], various home remedies including human and cow urine, kerosene, fuel and crude oil are used in cases of infantile cases of febrile convulsion [2,25,26].

In this study, apart from the viable and culturable Gram-positive bacterial species (Bacillus and Staphylococcus), the most prevalent Gram-negative bacterial species obtained from the children urine were E. coli (36.4%) and Proteus (18.2%), although other Gram-negative bacterial uropathogens, Citrobacter, Salmonella, Klebsiella, Pseudomonas and Shigella were also isolated, in agreement with previous studies on the aetiology of pediatric uropathogens [9,13,14,16,31-33]. Citrobacter, Klebsiella, Proteus, E. coli and Shigella were also the most recovered bacterial species from urine of cows in this study. These groups of pathogens are also similar to those obtained from some earlier studies [12,34,35]. Recovery of these group of indicator bacteria from urine that are consumed for the cure of clinical conditions in children is therefore, of great concern, especially because the isolated bacterial pathogens have been implicated in infantile and children gastroenteritis in some earlier studies [36-42]; and it is a well known fact that diarrhoeal diseases are a principal cause of childhood morbidity and mortality in the developing countries like Nigeria, being responsible for death of more than 4 million children each year [43-45].

It has also been well reported that antibiotic resistance demonstrates considerable geographic variability [31,45,47], while studies on pediatric uropathogens in most countries also indicated a rise in resistance to common antibiotics and continuing evolution of resistance to antimicrobial agents, as well as large inter-regional variability [9,13,31-33,48-50]. Moderate to high resistance (26-63%) to ampicillin, amoxicillin-clavulanic acid, cephalothin, cefuroxime and trimethoprim-sulfamethoxazole (SMZ/TMP) was noted among some of the bacteria in this study, which is similar to some earlier studies [13,33,51,52]. In the study of Pape et al. [11] as well, resistance rates to cotrimoxazole and 1st generation cefalosporines increased by about 20% compared with the previous analyses undertaken between 1990 and 1995, and therefore, concluded that the policies for treatment of UTI in children should be re-evaluated every 5 years according to local resistance rates. As an example, SMZ/TMP is a popular antibiotic in the treatment of pediatric UTIs and other clinical cases but the Infectious Disease Society of America had stated that with an SMZ/TMP resistance of 10–20% in adults, alternative first-line antimicrobial agents should be used [53]. This is based on adult uropathogen data but it is difficult to determine whether this same cut-off can be used in paediatric populations. Higher resistance (17.9-100%) to SMZ/TMP in this study therefore, raises some concerns.

Most of the bacteria isolated from urine of cows in this study also exhibited high rates of MAR towards 4 or more number of antibiotics. Globally, an estimated 50% of all antimicrobials serve veterinary purposes and literature of the last few years provides ample evidence that antibiotic resistance traits have entered the microflora of farm animals and the food produced from them [54]. Antimicrobial resistance has also emerged in zoonotic enteropathogens, commensal bacteria and bacterial pathogens of animals, although the prevalence of resistance varies [46,47,54]. In this present study, the recorded resistance rates as high as 87.5-100% observed among the bacterial species from urine of children, in spite of the fact that the bacterial species were non-UTI confirmed pathogens signifies that the introduction of strains that are drug resistant into a community plays a greater role in changing the prevalence of drug-resistant UTI [55]. In the five-year retrospective study by Ladhani and Gransden [55], bacterial isolates from children with underlying renal problems were generally more resistant to commonly used antibiotics in comparism with the children in the community, therefore, if such high antibiotic resistance (87.5-100%) as reported in this study were recorded among non-UTI confirmed bacterial strains, it then means that usage of urine of children and cows as alternative therapy in pediatric ill-health conditions like convulsion is quite hazardous.

Children are the most vulnerable members of any society since their immunity is not fully developed [45] but though the premium placed on children in African societies is so high, yet, even in the 21st century, the childhood mortality rates in the majority of African countries remain disturbingly high, with some 12 million children under–5 dying every year [56]. In 2006, there were 41 countries in which at least 10% of children under five died and all but three of the countries were in Africa. Ten of the 41 countries had higher rates of child mortality than 1990 and four were exactly the same. Among the worst 20, Nigeria ranked 12th with 181 deaths per 1000 [57]. Though the expected benefits of medical intervention should outweigh the possible harm, parents and guardians occasionally adopt some interventions which are futile, harmful and with no apparent curative or pathophysiologic rationale [25,58,59]. In Nigeria, as in most other developing countries, children are subjected to unorthodox treatment as first aid therapy in emergency conditions at home. In a study by Iyun and Tomson [60] in Nigeria, the reported dominating practice of mothers in cases of acute respiratory infections of children was either the use of irritants to get rid of the cause of the disease (‘coldness’) through vomiting, by forcing the child to swallow bitter remedies such as cow urine, or to use a remedy with warming and soothing properties.

## Conclusion

Urine therapy is being advocated worldwide as an alternative therapy in many clinical cases. This study however, confirms that there could be introduction of multiple antibiotic resistant bacterial pathogens through consumption of cow and human urine in pediatric cases, more especially, since urine has not been reported to be of any medical benefit in cases of convulsion or other pediatric health condition. Although the magnitude of antibiotic resistance vary among regions but the rates are alarmingly higher in the developing countries. This study has been able to highlight the public health implications of urine as alternative therapy in paediatric convulsion in a developing country like Nigeria, which is of great concern. Only the non-fastidious, viable and culturable bacterial species were isolated in this study, indicating that more pathogenic strains can be isolated with more sophisticated culture media and kits. It is strongly suggested that alternative therapies should be non-hazardous, and therefore, inappropriate administration of remedies, such as urine therapy in pediatric health conditions should be discouraged, considering the fact that no documented scientific / clinical evidence of the beneficial effect of urine therapy in clinical had been reported, while multiple antibiotic resistant bacterial species had also been recovered from such urine.

## Competing interests

The authors declare no competing interest in the study.

## Authors' contribution

V.A. Ajayi collected urine specimens from human subjects at Abeokuta locations and carried out some preliminary studies on the specimens. A.O. Fawole collected urine specimens from human subjects and cows at Ibadan locations, carried out some advanced studies on the specimens and wrote the draft of the results. A.A.O. Ogunshe supervised the collection of urine specimens at Abeokuta and Ibadan locations, carried out advanced studies on the specimens, corrected and edited the final write up of the manuscript.

## Acknowledgements

We are grateful to the fund raising associations “Tu ed io insieme” and “Oltre le parole” for their financial support, to the people and health center staff of Kyeibuza for kindly accepting to participate, Fr. Paolino Tomaino for his moral support, to Mr. Sanjeev Dani of Ranbaxy Laboratories Limited for provision of the vitamin tablets and Prof. Paoluzi for study review and precious advices.

## References

[1] Dinulos JG, Graham EA (1998). Influence of culture and pigment on skin conditions in children. Pediatr Rev..

[2] Otaigbe BE, Adesina AF (2005). Crude oil poisoning in a 20-year old Nigerian - a case report. Anil Aggrawal´s Intern J Forens Med Toxicol..

[3] Kayserili E, Unalp A, Apa H, Asilsoy S, Hizarcioglu M, Gulez P, Agin H (2008). Parental knowledge and practices regarding febrile convulsion. Turk J Med Sci..

[4] National Institute of Health Consensus Statement (1980).

[5] Kafaru E Urine therapy – another simple cure.

[6] Shodipe A (1986). Traditional treatment for hypertension, stroke, asthma, sickle cell, anemia, small pox and diabetes. The state of medical plants in Nigeria.

[7] Adodo (2001). Nature Power.

[8] Pasteur L (1963). Comptes rend us hebdomadaires des séances de l’academie des sciences.

[9] Rajkumar S, Saxena Y, Rajagopal V, Sierra MF (1988-1989). Trimethoprim in pediatric urinary tract infection. Child Nephrol Urol..

[10] Newman TB, Bernzweig JA, Takayama JI, Finch SA, Wasserman RC, Pantell RH (2002). Urine testing and urinary tract infections in febrile infants seen in office settings: the Pediatric Research in Office Settings’ Febrile Infant Study. Arch Pediatr Adolesc Med..

[11] Pape L, Gunzer F, Ziesing S, Pape A, Offner G, Ehrich JH (2004). Bacterial pathogens, resistance patterns and treatment options in community acquired pediatric urinary tract infection. Klin Padiatr.

[12] Yeruham I, Elad D, Avidar Y, Goshen T, Asis E (2004). Four-year survey of urinary tract infections in calves in Israel. Vet Rec.

[13] Gaspari RJ, Dickson E, Karlowsky J, Doern G (2005). Antibiotic resistance trends in pediatric uropathogens. Int J Antimicrob Agents.

[14] Akram M, Shahid M, Khan AU (2007). Etiology and antibiotic resistance patterns of community-acquired urinary tract infections in J N M C Hospital Aligarh, India. Ann Clin Microbiol Antimicrob.

[15] Zhanel GG, Hisanaga TL, Laing NM, DeCorby MR, Nichol KA, Palatnick LP, Johnson J, Noreddin A, Harding GK, M Nicolle LE, Hoban DJ (2005). Antibiotic resistance in outpatient urinary isolates: final results from the North American Urinary Tract Infection Collaborative Alliance (NAUTICA). Int J Antimicrob Agents.

[16] Kumar MS, Lakshmi V, Rajagopalan R (2006). Occurrence of extended spectrum beta-lactamases among Enterobacteriaceae spp isolated at a tertiary care institute. Indian J Med Microbiol.

[17] McNulty CA, Bowen J, Clark G, Charlett A, Cartwright K, South West G (2004). Microbiology Laboratory Use Group: How should general practitioners investigate suspected urinary tract infection? Variations in laboratory-confirmed bacteriuria in South West England. Commun Dis Public Health.

[18] National Committee of Clinical Laboratory Standards (1999). Performance standards for antimicrobial susceptibility testing: ninth international supplement.

[19] National Committee of Clinical Laboratory Standards (2003). Performance standards for antimicrobial disk susceptibility tests.

[20] Ogunshe AAO, Olaomi JO (2008). In-vitro phenotypic bactericidal effects of indigenous probiotics on bacterial pathogens implicated in infantile bacterial gastroenteritis using Tukey-HSD test. Amer J Infect Dis..

[21] Tagg JR, Dajani AS, Wannamaker LW (1976). Bacteriocins of Gram-positive bacteria. Bacteriol Rev.

[22] Osuntokun BO (1969). Convulsive disorders in Nigerians: the febrile convulsions (An evaluation of 155 patients). East Afr Med J.

[23] Vestergaard M, Pedersen MG, Østergaard JR, Pedersen CB, Olsen J, Christensen J (2008). Death in children with febrile seizures: a population-based cohort study. Lancet.

[24] Nwokocha EE, Awomoyi AO (2009). Factors influencing mothers´ role in convulsion treatment among under-five children in Ibadan, Nigeria. World Health Popul.

[25] Okoji GO, Peterside IE, Oruamabo RS (1993). Childhood convulsions: a hospital survey on traditional remedies. Afr J Med Med Sci.

[26] Anochie I, Graham-Douglas IB (2000). Non-accidental injuries associated with convulsions in Port Harcourt, Nigeria. Anil Aggrawal Internet Journal of Forensic Medicine and Toxicology.

[27] Ofovwe GE, Ibadin OM, Ofovwe EC, Okolo AA (2002). Home management of febrile convulsion in an African population: a comparison of urban and rural mothers´ knowledge, attitude and practice. J Neurol Sci.

[28] Nelson KB, Ellenberg JH (1976). Predictors of epilepsy in children who have experienced febrile seizures. N Engl J Med.

[29] Verity CM, Butler NR, Golding J (1985). Febrile convulsions in a national cohort followed up from birth, I—prevalence and recurrence in the first five years of life. Br Med J (Clin Res Ed).

[30] Verity CM, Golding J (1991). Risk of epilepsy after febrile convulsions: a national cohort study. BMJ.

[31] al-Mugeiren MM, Qadri SM (1996). Bacteriologic profile and drug resistance in pediatric patients with symptomatic bacteriuria. Clin Ther.

[32] McLoughlin TG, Joseph MM (2003). Antibiotic resistance patterns of uropathogens in pediatric emergency department patients. Acad Emerg Med.

[33] Zhanel GG, Hisanaga TL, Laing NM, DeCorby MR, Nichol KA, Weshnoweski B, Johnson J, Noreddin A, Low D, Karlowsky J (2006). Antibiotic resistance in Escherichia coli outpatient urinary isolates: final results from the North American Urinary Tract Infection Collaborative Alliance (NAUTICA). Int J Antimicrob Agents.

[34] Dewes HF, Goodall G (1995). Some preliminary observations on the possible relationship between ammonia production from soiled bedding in calf rearing sheds and calf illness. N Z Vet J.

[35] Wagenaar J, Zuerner RL, Alt D, Bolin CA (2000). Comparison of polymerase chain reaction assays with bacteriologic culture, immunofluorescence, and nucleic acid hybridization for detection of Leptospira borgpetersenii serovar hardjo in urine of cattle. Amer J Vet Res.

[36] Rokoszewska J, Smykal B, Bogdanowiez (1980). Mass poisoning with stew contaminated with Pseudomonas aeruginosa. Rocz Panstw Zakl Hig.

[37] Jiwa SF, Krovacek K, Wadström T (1981). Entertoxigenic bacteria in food and water from an Ethiopian community. Appl Environ Microbiol.

[38] Sears CL, Kaper JB (1996). Enteric bacterial toxins: Mechanisms of action and linkage to intestinal secretion. Microbiol Rev.

[39] Tirado C, Schmdit K (2001). WHO surveillance program for control of food borne infections and intoxications: results and trends across greater Europe. J Infect.

[40] Centers for Disease Control and Prevention (2002). Summary of notifiable diseases – United States. 2000. MMWR Morb Mortal Wkly Rep.

[41] Phelps KJ, Mckillip JL (2002). Enterotoxin production in natural isolates of Bacillaceae outside the Bacillus cereus as a cause of abortion in a mare. Current Science.

[42] Chen S, Zhao S, White DG, Schroeder CM, Lu R, Yang H, McDermatt PF, Ayers S, Meng J (2004). Characterization of multiple-antimicrobial-resistant Salmonella serovars isolated from retail meats. Appl Environ Microbiol.

[43] Anonymous A manual for the treatment of diarrhea. Control of diarrhoeal disease.

[44] Nader de Macias ME, Apella MC, Romero NC, Gonzalez SN, Oliver G (1992). Inhibition of Shigella sonnei by Lactobacillus casei and Lactobacillus acidophilus. J Appl Bacteriol.

[45] Lucas AO, Gilles HM Short Textbook of Public Health Medicine for the Tropics.

[46] Aarestrup FM, Bager F, Jensen NE, Madsen M, Meyling A, Wegener HC (1998). Resistance to antimicrobial agents used for animals therapy in pathogenic-, zoonotic- and indicator bacteria isolated from different food animals in Denmark: a baseline study for the Danish Integrated Antimicrobial Resistance Monitoring Programme (DANMAP). APMIS.

[47] Teuber M (2001). Veterinary use and antibiotic resistance. Curr Opin Microbiol.

[48] Gur D, Kanra G, Ceyhan M, Seçmeer G, Kanra B, Kaymakoğlu I (1999). Epidemiology and antibiotic resistance of Gram-negative urinary pathogens in pediatric patients. Turk J Pediatr.

[49] Huang I-F, Wagener MM, Hsieh K-S, Liu Y-C, Wu T-C, Lee W-Y, Chiou CC (2004). Nontyphoid salmonellosis in Taiwanese children: clinical manifestations, outcome and antibiotic resistance. J Pediatr Gastroenterol Nutr.

[50] Ogunshe AAO, Kolajo TT (2006). In vitro phenotypic antibiotic resistance in bacterial flora of some indigenous orally consumed herbal medications in Nigeria. J Rural Trop Publ Health.

[51] Mache A (2001). Antibiotic resistance and sero-groups of shigella among paediatric out-patients in southwest Ethiopia. East Afr Med J.

[52] Yüksel S, Oztürk B, Kavaz A, Özçakar Z, Acar B, Güriz H, Aysev D, Ekim M, Yalçınkaya F (2006). Antibiotic resistance of urinary tract pathogens and evaluation of empirical treatment in Turkish children with urinary tract infections. Int J Antimicrob Agents.

[53] McCarty JM, Richard G, Huck W, Tucker RM, Tosiello RL, Shan M, Heyd A, Echols RM (1999). A randomized trial of short-course ciprofloxacin, ofloxacin, or trimethoprim/sulfamethoxazole for the treatment of acute urinary tract infection in women: Ciprofloxacin Urinary Tract Infection Group. Am J Med.

[54] McEwen SA, Fedorka-Cray PJ (2002). Antimicrobial use and resistance in animals. Clin Infect Dis.

[55] Ladhani S, Gransden W (2003). Increasing antibiotic resistance among urinary tract isolates. Arch Dis Child.

[56] UNICEF (2008). Releasing declining numbers for child mortality, UNICEF calls for increased efforts to save children’s lives.

[57] UNICEF http://www.unicef.org/infobycountry/index.html.

[58] Fagbule D, Chike-Obi U D, Akintunde EA (1991). Febrile convulsions in Ilorin. Niger J Paediatr.

[59] Angyo I. A, Lawson JO, Okpeh ES (1997). Febrile convulsions in Jos. Niger J Paediatr.

[60] Iyun BF, Tomson G (1996). Acute respiratory infections - mothers´ perceptions of etiology and treatment in South-Western Nigeria. Soc Sci Med.

